# Evaluation of burnout and stress perception levels of Turkish dental laboratory technicians according to affecting factors during the COVID-19 pandemic

**DOI:** 10.1080/07853890.2023.2184487

**Published:** 2023-03-09

**Authors:** Sibel Dikicier, Cumhur Korkmaz, Arzu Atay, Mert Dogukan Yilmaz

**Affiliations:** a Department of Prosthodontics, University of Health Sciences, Istanbul, Turkey; b Dental Clinician, Istanbul, Turkey

**Keywords:** COVID-19, dental healthcare, burnout, sense of coherence, perceived stress

## Abstract

**Background:**

The coronavirus disease-2019 (COVID-19) pandemic has caused healthcare professionals to face unequal acute workplace stress and burnout. This study aimed to analyze the potential impact of COVID-19 on the burnout and associated emotional stress conditions of Turkish dental technicians.

**Methods:**

A 20-question demographic scale, Maslach Burnout Inventory (MBI), Sense of Coherence-13 (SoC-13), and Perceived Stress Scale-10 (PSS-10) were used to obtain data. A total of 152 participants answered these surveys directly and reported their stress burnout levels during the COVID-19 pandemic.

**Results:**

Of all participants who agreed to participate in the survey, 39.5% were females and 60.5% were males. Regardless of demographic variables, the MBI-total (37.2 ± 11.71), SoC-13 total (53.81 ± 10.29), and PSS-10 total (21.25 ± 5.5) scores indicated moderate burnout, SoC, and perceived stress levels. According to sub-scores of the MBI; mean emotional exhaustion and depersonalization indicate low-level burnout, and mean personal accomplishment indicates moderate burnout. Long working hours increase burnout. No significant differences were observed according to demographic variables, except for work experience. A positive correlation was found between perceived stress and burnout.

**Conclusions:**

The findings showed that dental technicians working during the COVID-19 pandemic are influenced by emotional stress due to the outcomes of the pandemic. One reason for this situation might be the long working hours. Working arrangements, under-controlled disease risk factors, and lifestyle changes may improve stress levels.

**Key Messages**
- COVID-19 outbreak exposed psychological returns to the general population, and especially to healthcare workers.
- Questionnaire method was applied to evaluate the burnout and stress levels among dental laboratory technicians during COVID-19 outbreak.
- Moderate levels of burnout and stress perception were detected. Long working time was one of the effective factors.

## Background

In December 2019, a novel coronavirus, which causes pneumonia, was identified in China, and the World Health Organization named this virus ‘severe acute respiratory syndrome coronavirus-2’ (SARS-CoV-2) [1,2]. At the international level, following the acceptance of coronavirus disease-2019 (COVID-19) as a global pandemic, the focus was primarily on providing emergency health services. After the official announcement of the pandemic, pandemic strategies were rapidly planned, and the COVID-19 guidelines were published in Turkey and announced to all healthcare professionals. Possible case definitions and case management, use of protective equipment by individuals and healthcare professionals, follow-up of people in contact with possible COVID-19 cases, and precautions to be taken were specified [2–4].

In particular, due to cross-infection, oral and dental healthcare services are considered high risk for contamination by blood or other body fluids, airborne infectious agents, and aerosols [3]. Furthermore, SARS-CoV-2 can remain as aerosols for up to 3 h and on plastic and steel surfaces for 2–3 d. For this reason, it is suggested that keeping the dental equipment and surrounding environment clean and dry will reduce the adhesion of the virus [2,5].

In the face of the COVID-19 pandemic, healthcare professionals worldwide are under extraordinary stress risk factors. Among them; excessive workload, insufficient knowledge about the mechanism of the disease, an insufficient supply of personal protective equipment, fear of being infected, or infecting their families and relatives are remarkable. Mental health problems adversely affect the ability of healthcare professionals to make the right decision and carry out treatment processes [6]. A previous study in China showed that some mental disorder results in healthcare professionals were 50%, 45% and 34% for depression, anxiety, and insomnia, respectively [5].

It is important that the management of the pandemic and the following process be carefully planned for all branches of oral and dental health services within the scope of common measures. However, relevant data show that this occupational group had a higher risk of exposure among all healthcare services [7]. This situation indirectly creates additional stress factors for dentists and staff involved in dental practice, such as dental technicians and assistants [8].

Among oral and dental health professionals, dental laboratory technicians are at a prominent risk of exposure to SARS-CoV-2. Aerosols, contaminated laboratory surfaces, indirect contact with dental clinics through impressions, dental casts, and various prosthetic equipment may constitute that risk. Dental health workers may be subjected to social isolation, anxiety, and fatigue, in addition to extreme work stress under pandemic conditions. Working in high-risk areas and providing treatment services to infected patients may cause further traumatic effects [9].

All these negativities of the pandemic may lead to burnout among healthcare professionals, and it is worried that posttraumatic stress disorder may become a ‘parallel epidemic’ [10]. Burnout is an issue that has been frequently discussed before COVID-19. However, because healthcare professionals are among the groups that take great responsibility during the pandemic period, burnout is emphasized in this period. Varying levels of burnout are observed among healthcare professionals, depending on their specialty, work hours, and personal characteristics [11,12].

Although many studies on the effects of the COVID-19 pandemic on perceived stress and burnout levels of dental health professionals are primarily reported for dental students, little is known about the burnout and stress conditions of dental technicians [13–17]. This study aimed to investigate dental technicians’ health risk concerns, impacts on work performance, and emotional conditions during the COVID-19 outbreak using the questionnaire method. It also aimed to explore the relationship between each demographic variable and the symptoms of burnout, sense of coherence and perceived stress. The null hypothesis of this research indicate that the pandemic has created a high level of burnout and that there is a relationship between burnout, SoC, and perceived stress.

## Methods

### Study design

The descriptive cross-sectional study population consisted of dental technicians who actively worked in private or public institutions during the pandemic in Istanbul, Turkey. It was considered the total population size (*n* = 300) and at a 95% confidence interval with a 0.05 margin of error in the calculated sample size (*n* = 169) using a simple random sampling method. Finally, 170 questionnaires were distributed, and 152 were completed by obtaining a response rate of 89%.

The inclusion criteria were as follows: (a) aged ≥18 years; (b) taking part in the relevant oral and dental health practice during the COVID-19 pandemic; (c) having an adequate level of cognitive ability and literacy to complete the questionnaire; (d) being informed by documents and presentations about COVID-19 published by professional organizations during the pandemic, including the principles of oral and dental health services and preventive measures to be provided in public/private institutions; and (e) volunteering to participate in the study. The exclusion criteria were as follows: (a) not taking part in the relevant oral and dental health practice during the COVID-19 pandemic; (b) insufficient cognitive ability and inadequate level of literacy to complete the questionnaire; (c) not being informed by the documents and presentations mentioned above; and (d) not volunteering to participate in the study.

A demographic questionnaire was recorded, including the sociodemographic status and working background of the participants, before being recruited into the research. Accordingly, participants who met the inclusion criteria signed a consent form that included information about the purpose of the study and the conditions of participation. Participants with incomplete or missing information and those who did not provide their consent form were not included. Paper-formed questionnaires were distributed to participants by an assistant researcher between January and April 2021, it was also provided that the participants with technical information about the response procedures.

### Data collection

A 20-question demographic scale, the Turkish version of the Maslach Burnout Inventory (MBI), consisting of 22 questions developed by Maslach & Jackson [18], Antonovsky’s Sense of Coherence-13 (SoC-13), and Perceived Stress Scale-10 (PSS-10) were used as data collection tools.

#### Maslach burnout inventory-human services survey (MBI-HSS)

Previous investigationd about the psychometric properties of the MBI have been limited. However, the ‘MBI has been tested among healthcare professionals and has demonstrated a consistent three-factor structure [18–20].’

The survey contained 22 items with three dimensions: emotional exhaustion (EE, 9 items), depersonalization (DP, 5 items), and personal accomplishment (PA, 8 items). Each item is measured on a five-point Likert scale. The measurement of the survey is made as ‘Never = 0’ and ‘Always = 4.’ Scores from 0 to 32 can be obtained on the scale for the PA subscale, 0–20 for the DP and 0–36 for the EE. The burnout level of Turkish dental technicians was used to analyze the prevalence of the following subdimensions in this study: EE (low <18, moderate 18–19, high >22), depersonalization (low <10, moderate 10–11, high >13), and PA (low <16, moderate 16–17, high >20). For the EE and DP subscales, higher scores signaled intense burnout, while for the PA subscale, lower scores indicated intense burnout [18]. Cronbach’s alpha was 0.87 in the current study.

#### Sense of coherence-13 (SoC-13)

According to Antonovsky [21], SoC defines how individuals can manage the diversity of stress factors in daily life. This view focuses on factors that encourage and protect well-being and resources such as capabilities and efficiencies [22]. The version used here was the SoC-13. Each item is scored on a scale of 1–7, with a total range of 13–91 points. A higher score indicates a powerful SoC, but no reference score has been reported in the literature [23]. Cronbach’s alpha was 0.86 in the current study.

#### Perceived stress scale-10 (PSS-10)

The PSS-10 is a 10-item scale evaluated to assess self-perceived stress. Each item was scored as 0, never; 1, almost ever; 2, sometimes; 3, fairly often; and 4, very often on a 5-point Likert scale. The scale forms two domains: six positively (items 1, 2, 3, 6, 9, and 10) and four negatively (items 4, 5, 7 and 8) that require reversion-worded items. Total scores range from 0 to 40, with higher scores indicating higher levels of perceived stress [24,25]. Cronbach’s alpha was *α* = 0.86 in the current study.

### Statistical methods

The total scores of MBI, SoC-13, and PSS-10 were analyzed, and their relevant descriptive measures (mean and standard deviation [SD]) were calculated. For analysis purposes, these scores were considered continuous variables. Data analyses were conducted using IBM SPSS Statistics version 20 (NY, USA). The analysis of variance test (post hoc: Bonferroni) was used to determine the comparisons between groups and the z-test was used to compare two different means. Statistical significance was set at *p* < .05. The correlation analysis was performed with Pearson’s correlation using an entry procedure with the dependent variables severe MBI, SoC-13, and PSS-10.

## Results

A total of 152 (98.7%) participating dental technicians were included in the cross-sectional analysis, with consideration of completed surveys. Table 1 shows the participation rates of the demographic variables included in the study. While 56.6% of the participants were aged <35 years and 15.8% were >45 years. Of these, 60.5% were males and 39.5% were females. No significant differences were detected in the income levels of the participants. In terms of educational level, associate degree graduates represented the majority, with 93 participants (61.2%). The most important demographic data in this study were work experience and working hours. The proportion of participants with 15–25 years of working experience was higher in the total study. The working hours of all participants were similar.

**Table 1.** Characteristics of study participants (*n* = 152).

Characteristics	Participants (*n*)	Percentage (%)
Age (year)		
20–24	35	23.0
25–34	51	33.6
35–44	42	27.6
45+	24	15.8
Gender		
Male	92	60.5
Female	60	39.5
Marital status		
Single	52	34.2
Divorced	7	4.6
Married	93	61.2
Income level (TL)		
1500–2500	23	15.1
2600–3500	63	41.4
3600+	66	43.4
Education		
High school	53	34.9
Associate degree	93	61.2
Graduate/Postgraduate	6	3.9
Working experience (year)		
1–5	41	27.0
5–10	21	13.8
10–15	37	24.3
15–25	53	34.9
Working time (weekly-hour)		
40	55	36.2
50	48	31.6
60	49	32.2

Regardless of demographic characteristics, the total mean scores of the Turkish versions of MBI, SoC-13, and PSS-10 were analyzed. According to the total burnout and subdimension scores, the total burnout score indicates moderate burnout (37.2 ± 11.71), mean EE (13.04 ± 7.57) and DP (4.43 ± 4.29) indicate low-level burnout and mean PA sub-score (19.55 ± 6.83) indicates moderate burnout.

The highest total MBI (highest total MBI score) was associated with the highest levels of total burnout. The MBI-total and subgroup scores did not reveal large differences in age, marital status, income level, and education groups. The same findings were found in the analysis by gender, but statistically significant differences between females and males were found in terms of DP scores (*p* < .05).

Based on the burnout analysis, a significant difference was observed with the participant’s background in working experience and weekly working time. The MBI-total and subsequent subgroup scores of the participants with a working period of 5–10 years (EE,17.76 ± 8.1; DP, 7.05 ± 4.1; MBI total, −46.29 ± 13.38) were significantly higher than those with a working period of 10–15 years and 15–25 years (*p* < .05). Similarly, these results indicated that the burnout levels of participants with a working period of 5–10 years increased more than those of the other groups.

It was also observed that the ‘working time weekly’ parameter also affected burnout scores. Statistically significant differences were found between scores in the MBI subgroups. Those who worked 40 h had lower EE and DP scores and higher PA scores than those who worked 50 and 60 h weekly. These findings highlight that increased working hours increase burnout levels. Details of the above-mentioned sources of information are presented in Table 2.

**Table 2.** Association of variables of study participants with burnout levels of MBI.

Characteristics	Emotional exhaustion	Depersonalization	Personal accomplishment	MBI-total
Age (year)	Mean (SD)	Mean (SD)	Mean (SD)	Mean (SD)
20–24	13.09 (7.31)	4.43 (4.13)	18.8 (7.19)	36.31 (11.96)
25–34	12.92 (7.83)	4.2 (3.95)	20.39 (6.88)	37.51 (11.79)
35–44	14.69 (7.86)	5.14 (4.8)	19 (7.59)	38.83 (12.82)
45+	10.33 (6.42)	3.71 (4.35)	19.79 (4.62)	33.83 (8.71)
p_*_	.17	.58	.69	.39
Gender				
Male	13.48 (8.07)	4.98 (4.53)^a^	19.68 (7.05)	38.14 (12.78)
Female	12.37 (6.74)	3.6 (3.77)^a^	19.33 (6.54)	35.3 (9.7)
p_*_	.36	.04^**^	.76	.14
Marital status				
Single	13.88 (7.41)	4.25 (4.05)	18.73 (7.09)	36.87 (11.37)
Divorced	14.29 (8.75)	5.71 (7.04)	19.57 (5.06)	39.57 (14.68)
Married	12.47 (7.6)	4.44 (4.21)	20 (6.82)	36.91 (11.79)
p_*_	.51	.70	.57	.84
Income level (TL)				
1500–2500	13.96 (8.67)	4.96 (5.08)	18.35 (4.84)	37.26 (12.86)
2600–3500	13.73 (7.47)	4.81 (4.06)	18.95 (7.47)	37.49 (12.21)
3600+	12.06 (7.26)	3.89 (3.65)	20.53 (6.74)	36.48 (10.95)
p_*_	.38	.39	.28	.88
Education				
High school	14 (7.17)	5.3 (4.67)	18.15 (7.58)	37.45 (13.56)
Associate degree	12.44 (7.86)	4.05 (4.06)	20.03 (6.32)	36.53 (10.77)
Graduate/Postgraduate	13.83 (6.52)	2.67 (3.27)	24.33 (4.84)	40.83 (8.57)
p_*_	.48	.14	.06	.65
Working experience (year)				
1–5	12.95 (7.33)^b^	3.66 (3.71)^c^	19.46 (6.9)	36.07 (8.83)^d^
5–10	17.76 (8.1)^b^	7.05 (4.1)^c^	21.48 (7.27)	46.29 (13.38)^d^
10–15	12.24 (7.5)^b^	3.22 (3.66)^c^	18.57 (7.14)	34.03 (12.39)^d^
15–25	11.79 (7.06)^b^	4.85 (4.75)^c^	19.53 (6.41)	36.17 (10.99)^d^
p_*_	.02^**^	.001^**^	.49	.001^**^
Working time (weekly-hour)				
40	10.71 (6.38)^e^	2.98 (3.29)^f^	21.29 (6.12)^g^	34.98 (10.9)
50	14.19 (6.99)^e^	4.38 (4.02)^f^	18.15 (6.86)^g^	36.71 (10.35)
60	14.53 (8.77)^e^	6.12 (4.95)^f^	18.97 (7.26)^g^	39.61 (13.47)
p_*_	.02^**^	.001^**^	.049^**^	.13

SD: Standart deviation; MBI: Maslach Burnout Inventory; TL: Turkish Liras.

*ANOVA (post hoc:Bonferroni) and z test (*p* < .05). “** significantly different” expression.

^a,b,c,d,e,f,g ^significantly difference among the groups (p<.05).

To investigate which variables impacted MBI scores, a Pearson’s correlation analysis was performed (Table 3). Overall, the weekly working hours were significant for the analysis. In both analyses, EE and DP scores significantly influenced working time, and increased education level had a positive effect on PA scores.

**Table 3.** Correlation analysis of the MBI subscores, MBI total, SOC-13, and PSS-10 related to the dependent variables.

		Age	Income level	Education	Working experience	Working time
Emotional exhaustion	*r* ^*^	−.037	−.101	−.093	−.109	.190^**^
	*p*	.652	.215	.255	.183	.019
Depersonalization	*r* ^*^	−.037	−.053	−.137	.015	.275^**^
	*p*	.654	.514	.093	.850	.001
Personal accomplishment	*r* ^*^	.028	.134	.170^**^	−.004	−.158
	*p*	.730	.100	.037	.958	.052
MBI-total	*r* ^*^	−.003	−.024	.032	−.056	.128
	*p*	.967	.767	.692	.497	.115
SoC-13	*r* ^*^	.024	.065	.088	.006	−.078
	*p*	.769	.427	.281	.944	.340
PSS-10	*r* ^*^	−.187^**^	−.019	.048	−.106	.025
	*p*	.021	.821	.555	.195	.755

*Spearman correlation coefficient.

**Statistically significant MBI: Maslach Burnout Inventory; SoC-13: Sense of Coherence-13; PSS-10: Perceived Stress Scale-10.

The mean scores of the Turkish versions of SoC-13 and PSS-10 for dental technicians and the demographic variables are presented in Table 4. All available independent variables were determined not to be significantly different from each other, based on SoC-13 scores. Participants with divorced marital status had the highest mean score for SoC-13 (58.29 ± 11.47), and the lowest score was in the income level group (52.25 ± 11.07), but the difference was not statistically significant (Table 4).

**Table 4.** The distribution of mean scores from the SOC-13 and PSS-10 in study characteristics.

Characteristics	SoC-13	PSS-10
Age (year)	Mean (SD)	Mean (SD)
20–24	54.54 (8.3)	22.83 (5.76)
25–34	53.18 (11.16)	21.27 (5.28)
35–44	53.02 (11.72)	20.64 (5.82)
45+	55.46 (8.54)	19.96 (4.71)
p*	.75	.19
Gender		
Male	53.11 (10.99)	20.9 (5.64)
Female	54.88 (9.12)	21.78 (5.27)
p_*_	.30	.34
Marital status		
Single	52.87 (10.01)	21.63 (5.24)
Divorced	58.29 (11.47)	19 (3.51)
Married	54 (10.38)	21.2 (5.75)
p_*_	.41	.49
Income level (TL)		
1500–2500	54.74 (8.96)	20.13 (5.72)
2600–3500	52.25 (11.07)	21.92 (5.9)
3600+	54.97 (9.9)	21 (5)
p_*_	.29	.37
Education		
High School	52.42 (9.61)	20.64 (5.84)
Associate degree	54.62 (10.52)	21.67 (5.45)
Graduate/Postgraduate	53.5 (13)	20.17 (1.72)
p_*_	.46	.50
Working experience (year)		
1–5	52.85 (8.46)	21.9 (5.49)
5–10	56.24 (11.89)	21.81 (5.06)
10–15	54.08 (10.62)	21.35 (5.83)
15–25	53.4 (10.82)	20.45 (5.49)
p_*_	.66	.59
Working time (weekly-hour)		
40	55.69 (10.6)	21.27 (5.19)
50	51.15 (9.13)	20.81 (5.75)
60	54.31 (9.96)	21.65 (5.66)
p_*_	.08	.76

SoC-13: Sense of Coherence-13; PSS-10: Perceived Stress Scale-10.

*ANOVA (post hoc:Bonferroni) and *z* test (*p* < .05).

In both analyses, PSS-10 did not significantly influence demographic variables, while PSS-10 had small positive significant correlations with age (Table 5). According to Pearson’s correlation, a strong positive correlation was found between EE and PSS-10 and MBI-total and PSS-10 scores (*p* < .05, *r* = 0.215; *p* < .05, *r* = 0.212, respectively) As EE and total MBI scores increased, PSS-10 scores also increased. Within the MBI subscores, a positive correlation was found only between the EE and DP scores (*p* < .001, *r* = 0.417). A correlation was also determined between the participants’ SoC and MBI subscores. Although EE scores had a negative effect, increased PA scores increased the success of stress management (Table 5).

**Table 5.** Correlation of the MBI subscores, MBI total, SoC-13 and PSS-10.

		EE	DP	PA	MBI-total	SoC-13	PSS-10
EE	*r*	1	.417**	−.069	.759**	−.206**	.215**
	*p*		.0001	.400	.0001	.011	.008
DP	*r*	.417**	1	−.088	.584*	−.127	.058
	*p*	.0001		.281	.0001	.120	.478
PA	*r*	−.069	−.088	1	.507**	.376**	.088
	*p*	.4001	.281		.000	.0001	.281
MBI-total	*r*	.759**	.584*	.507**	1	.040	.212**
	*p*	.0001	.0001	.0001		.628	.009
SoC-13	*r*	−.206*	−.127	.376*	.040	1	.103
	*p*	.011	.120	.0001	.628		.206
PSS-10	*r*	.215**	.058	.088	.212**	.103	1
	*p*	.008	.478	.281	.009	.206	

*Pearson correlation coefficient.

**Statistically significantly difference among the groups (p<.05).

EE: Emotional Exhaustion; DP: Depersonalization; PA: Personal Accomplishment.

## Discussion

In the current study, we investigated whether COVID-19 pandemic conditions can affect the burnout and stress levels of Turkish dental technicians, simultaneously analyzing with other demographic variables, income level, work experience, and time. Our main findings confirmed moderately high levels of burnout, SoC, and perceived stress during the COVID-19 pandemic. Age, gender, and marital status were not significantly associated with total MBI, SoC-13, and PSS-10. The analysis of Turkish reliability and validity of the questionnaires listed below has been reported in many previous studies [21,26–29].

Zerbini et al. [30] demonstrated that healthcare workers, especially those working in direct contact with patients with COVID-19, are at higher risk for psychological adversity compared to other colleagues. According to data in the literature, many studies on this subject are seen to be mainly focused on nurses [30–32]. However, as far as we know, no previous work has studied stress levels among dental laboratory staff that are not working front-line during a pandemic.

Demographic variables showed that long-term work experience and less working time per week represented protective variables for burnout. In addition, new professional participants showed more symptoms of pyschological distress. This result may be because participants with less work experience had lower control over processes and decision-making capacity than other experienced participants. According to many previous studies; there were the same results that less working experience of healthcare workers related to worse mental health outcomes similar to our study [33–35]. Jalili et al. [35] demonstrated an increase level of burnout in those with ≤5 years than >5 years of working experience. In contrast to our analysis, Zhu et al. [36] identified longer working experience >10 years as a risk factor for pyschological impact. This result could be possibly associated with that most healthcare workers with more than 10 working years had more occupational exhaustion and family responsibilities.

In conclusion, the primary signs of COVID-19-related outcomes (fear of contamination and stress at work) were related to general measures of burnout and stress levels, suggesting that the COVID-19 pandemic is an emotionally and physically stressful period. Many studies focusing on the emotional conditions of healthcare workers during the pandemic have reported high levels of burnout and stress [12,13,30,32,37]. Batista et al. [9] revealed that prosthodontists and dental technicians are at high risk of infection with COVID-19 through aerosols, contaminated surfaces, prosthodontic equipment, and prosthetic appliances. As the work time increases, the experience and self-confidence of the individual increase; therefore, this situation reflects positively on dental practice, which can be explained as reducing the level of burnout. In the current study, participants with the least work experience (5–10 years) had a higher level of burnout.

However, in contrast to other studies [38,39] we did not find a significant effect on our primary outcome measure. This could be due to a lower initial level of emotional burnout than expected in our sample, especially compared to previous studies on healthcare professionals. Additionally, a previous study has shown that dental technicians and laboratory staffs are not exposed to the same high levels of risk as clinicians. This result is explained by the fact that technicians work in a laboratory environment and therefore do not have physical contact with patients, interact less with other workers and are not subjected to direct contamination [40].

Turkish dental technicians have been negatively impacted due to their increased weekly working hours during the COVID-19 pandemic. According to the results, the levels of EE and DP were higher in technicians with more weekly working hours and less time to rest. Due to their long-time work status, healthcare professionals can increase stress perceptions and feelings that participation in the work is compulsory. As the COVID-19 outbreak can result in decreased enthusiasm to work, compulsory work may develop negative effects on psychological well-being in that controversy between work responsibilities and worry about contamination can affect mental health [41,42].

In a similar study supports our findings related to working conditions, Rapisarda et al. examined the burnout and mental health levels of mental health professionals. They reported that workplace conditions are a major risk for burnout symptoms. The authors also suggested that the role of adaptation to changes in work design in terms of workload and tasks due to the COVID-19 pandemic can be scaled back [43].

During the pandemic, there are also studies investigating other psychosocial parameters such as anxiety, insomnia, and mental health status in healthcare workers [9,44,45]. In this study, we evaluated parameters related to stress to establish a direct connection with burnout. SoC is considered an important guide to psychological well-being among individuals, indicating a source of self-protection when faced with stressful conditions [46,47]. However, we were interested in understanding the relationship between SoC and perceived stress and burnout during the pandemic, and especially how it works beyond specific working groups of healthcare. The initial analysis revealed significant differences between the participants in terms of SoC, marital status, and income level. Therefore, in this study, participants with moderate SoCs could understand the COVID-19 pandemic as an understandable, controllable, and significant situation. Similarly to our study, a previous study has shown that healthcare workers with low SoC know the effects of changing lifestyles compared with healthcare workers with moderate or potent SoC, as those with low SoC are at higher risk of poor mental health [45]. Because our results present significant interaction impacts between the SoC level and MBI subscores, we emphasize that an increase in SoC levels among individuals may be associated with a decrease in burnout.

The PSS-10 survey also focuses on certain groups at higher risk for psychological problems. In particular, the current finding of moderate to severe perceived stress levels in Turkish dental technicians, with a median PSS-10 score of 21. In this study, the prevalence of moderate perceived stress was relatively higher than in a previous study on Chinese health workers [48]. Meanwhile, the results of PSS-10 are similar to the findings of a study in the United Kingdom [38]. However, this study was conducted in general healthcare positions, including physicians, nurses, auxiliary health staff, and employees in management. The diversity in prevalence may be explained by the spectrum of symptoms explored by each study instrument and sample context. The correlational analysis showed that there were significant correlations between perceived stress, age, perceived stress, and MBI. Higher age usually indicated a higher perception of stress, and higher perceived stress levels triggered a high level of burnout during the COVID-19 pandemic.

### Limitations

This study had several limitations that should be considered when evaluating the results. A previous study emphasized that reducing burnout in healthcare workers is especially difficult [49]. However, despite the limited statistical effect sizes in this study, a few changes in burnout scores may not result in clinically significant differences. The response rate was relatively high and there may have been an undersampling of specific groups. Meanwhile, our study included self-reporting by participants, and there was a remarkable risk of response bias. Furthermore, this study did not determine the levels of depression, anxiety, and mental health. This should be explored further in future studies. Despite these limitations, the findings can provide information by instantly reflecting emotional stress levels during the pandemic in this occupational group.

## Conclusions

The current study demonstrated that burnout level, SoC, and perceived stress were significant factors associated with better psychological health among Turkish dental technicians during the working period during the COVID-19 pandemic. Furthermore, dental professionals, especially laboratory technicians in indirect contact with patients with COVID-19, are at risk of psychological return as much as other healthcare professionals. Based on the current findings, most of the participants reported moderate burnout, SoC, and perceived stress levels. This might be explained by the fact that they somewhat protected themselves from psychological effects. Furthermore, long working hours should be restricted to reduce burnout.

## Data Availability

The study data used to support the findngs of this study are available from the corresponding author upon request.
